# Data on the genome analysis of the wood-rotting fungus *Steccherinum ochraceum* LE-BIN 3174

**DOI:** 10.1016/j.dib.2020.105169

**Published:** 2020-01-22

**Authors:** Konstantin Moiseenko, Olga Glazunova, Natalia Shakhova, Olga Savinova, Daria Vasina, Tatiana Tyazhelova, Nadezhda Psurtseva, Tatiana Fedorova

**Affiliations:** a A.N. Bach Institute of Biochemistry, Research Center of Biotechnology, Russian Academy of Sciences, Leninsky Ave. 33/2, Moscow, 119071, Russian Federation; b Komarov Botanical Institute of the Russian Academy of Sciences, Professor Popov St. 2, St. Petersburg, 197376, Russian Federation; c N.I. Vavilov Institute of General Genetics, Russian Academy of Sciences, Gubkin St. 3, Moscow, 117809, Russian Federation

**Keywords:** *Steccherinum ochraceum*, Draft genome sequence, White-rot, Wood decay

## Abstract

In the present article, we report data on the whole-genome sequencing of wood-rotting (white-rot) fungus *Steccherinum ochraceum* LE-BIN 3174. The *S. ochraceum* LE-BIN 3174 genome consists of 770 scaffolds (N50 = 62,812 bp) with the total length of assembly ∼35 Mb. The structural annotation of the genome resulted in the prediction of 12,441 gene models, among which 181 were models of tRNA-coding genes, and 12,260 – protein-coding genes. The protein-coding genes were annotated with different databases (Pfam, InterPro, eggNOG, dbCAN, and MEROPS). The whole genome sequence and functional annotation provide an important information for the deep investigation of biochemical processes that take place during the late stages of wood decomposition by Basidiomycetes. The Whole Genome project of *S. ochraceum* LE-BIN 3174 had been deposited at DDBJ/ENA/GenBank under the accession RWJN00000000. The version described in this work is version RWJN00000000.1. For further interpretation of the data provided in this article, please refer to the research article “Fungal Adaptation to the Advanced Stages of Wood Decomposition: Insights from the *Steccherinum ochraceum*” [1].

**Table:** Specifications Table

Subject	Biology
Specific subject area	Microbiology, Mycology, Genomics.
Type of data	Genome sequence data.
How data were acquired	Shotgun method using Illumina HiSeq 2500 with paired end runs.
Data format	Raw and analyzed data.
Parameters for data collection	The mycelium derived from field-collected basidiospores was statically cultivated on glucose-peptone (GP) medium at 26–28 °C in 750-mL Erlenmeyer flasks. The mycelium was ground in liquid nitrogen, and total DNA was extracted using DNeasy Plant Mini Kit (Qiagen, US).
Description of data collection	The genome was assembled with CLC Genomics Workbench 11.0 (Qiagen, US) and annotated with Funannotate pipeline v1.5.0 (https://github.com/nextgenusfs/funannotate)
Data source location	The fungal strain of *Steccherinum ochraceum* (Pers. ex J.F. Gmel.) Gray was isolated (August 01, 2013) from basidiospores collected from a fallen dry aspen branch in the polydominant temperate deciduous broadleaf forest (Kaluzhskiye Zaseki Nature Reserve, Russia; N 53º33′28.4″; E 35º38′24.4″). The strain was deposited in the Komarov Botanical Institute Basidiomycetes Culture Collection (LE-BIN; St. Petersburg, Russia) as *S. ochraceum* LE-BIN 3174
Data accessibility	The whole genome sequence of *Steccherinum ochraceum* LE-BIN 3174 had been deposited at DDBJ/ENA/GenBank under the accession RWJN00000000. The version described in this paper is version RWJN00000000.1. The BioSample and BioProject accession numbers are SAMN10505049 and PRJNA507755, respectively. All other data are within this article.
Related research article	K.V. Moiseenko, O.A. Glazunova, N.V. Shakhova, O.S. Savinova, D.V. Vasina, T.V. Tyazhelova, N.V. Psurtseva, T.V. Fedorova, Fungal Adaptation to the Advanced Stages of Wood Decomposition: Insights from the *Steccherinum ochraceum*, Microorganisms. 7 (2019) 527. https://doi.org/10.3390/microorganisms7110527 [1].

**Table:** 

**Value of the Data** • The genome of *Steccherinum ochraceum* LE-BIN 3174 is the first genome under the family *Steccherinaceae* to be reported. • This draft genome will accelerate functional genomics research, increase the knowledge of the biochemical process of wood degradation and create an opportunity for comparative studies with other fungi. • The CAZyme content of this genome will provide a valuable insight into the fungal adaptation to an ecological niche of pre-degraded wood.

## 1 Data description

**Fig. 1:**
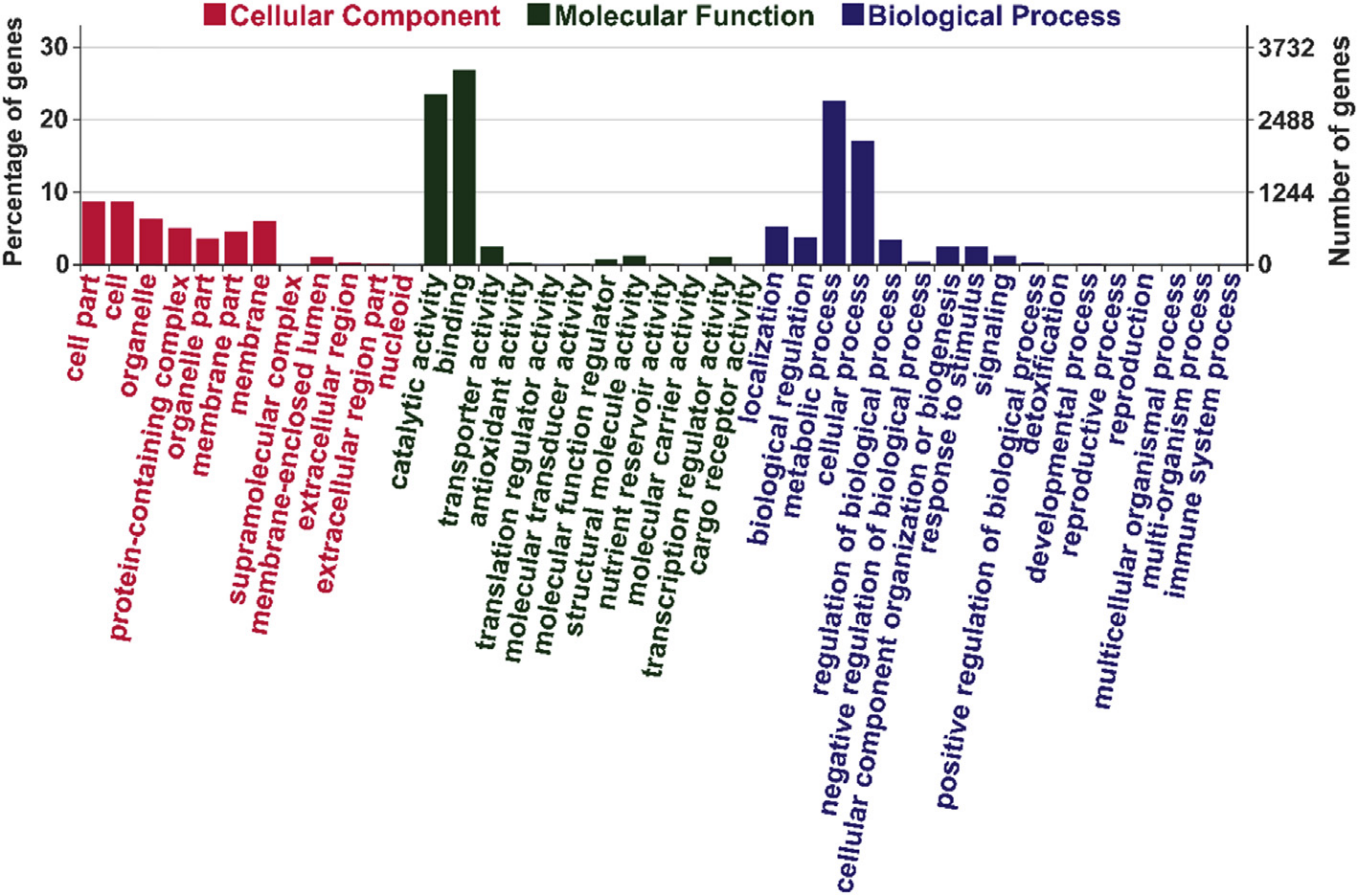
The Gene Ontology (GO) functional annotation of *S. ochraceum* LE-BIN 3174.

**Fig. 2:**
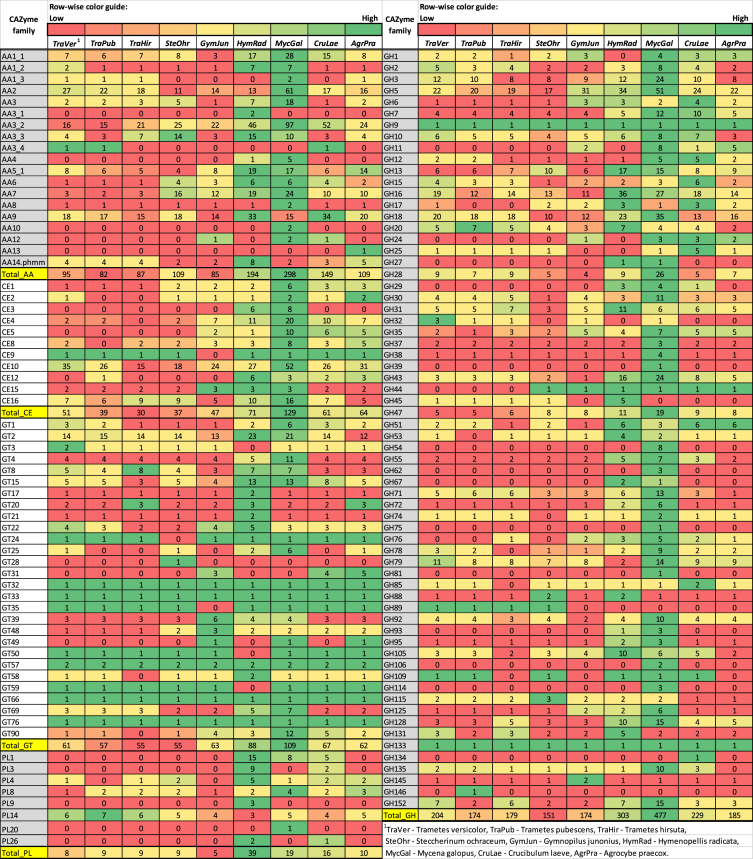
Families of carbohydrate-degrading enzymes (CAZymes) of *S. ochraceum* LE-BIN 3174 and other basidiomycetes.

**Fig. 3:**
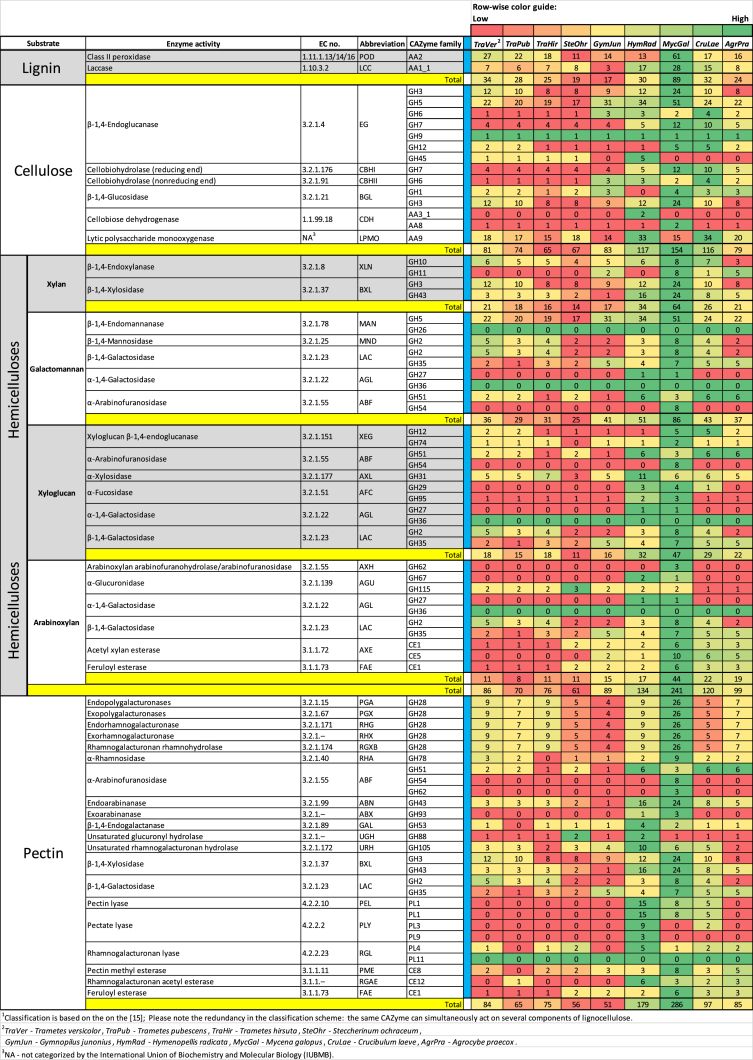
Families of carbohydrate-degrading enzymes (CAZymes) related to plant polysaccharide degradation in *S. ochraceum* LE-BIN 3174 and other fungal genomes.

*Steccherinum ochraceum* is a white-rot basidiomycete with wide ecological amplitude. It occurs in different regions of Russia and throughout the world occupying different climatic zones. The obtained draft genome of *S. ochraceum* LE-BIN 3174 (DDBJ/ENA/GenBank accession/version – RWJN00000000.1) is represented by the 770 scaffolds with the total length of 35.27 Mb and of comparable quality with other previously sequenced genomes of polypore fungi [2]. The gene prediction resulted in 12,441 gene models. The general information regarding genome's assembly, structural and functional annotation is presented in Table 1. The summary of the Gene Ontology (GO) classification of the protein coding genes is illustrated in Fig. 1. The whole genome sequence of *S. ochraceum* LE-BIN 3174 showed that it harbors 361 carbohydrate-active enzymes (CAZymes). The auxiliary activity enzymes (AA), carbohydrate esterase (CE), glycoside hydrolases (GH), glycosyl transferase (GT), and polysaccharide lyase (PL) superfamilies were represented by 109, 37, 151, 55, and 9 CAZymes from 9, 8, 48, 25, and 3 families, respectively. The comparison of the *S. ochraceum* CAZymes genome content with those from other lignocellulose decaying fungi belonging to different trophic groups is presented in Fig. 2 and Fig. 3.

**Table 1:** General data on the genome sequencing of *S. ochraceum* LE-BIN 3174.

Sequencing			
Sequencing technology	Illumina HiSeq 2500	Total number of paired-reads (2×)	2 × 47 868 586
Length of paired-reads (2×), bp	2 × 100	Insert size, bp	300–500
Assembly		Structural annotation	
Assembly size	35.27 (Mb)	Repeat content	1.4 (%)
Overall coverage	100×	Overall GC	52.7 (%)
Number of scaffolds	770	Number of predicted genes	12 441
Longest scaffold	464 123 (bp)	Proportion covered by genes	64.1 (%)
N50 length of scaffolds	62 812 (bp)	Number of tRNA-coding genes	181
Mean size of scaffolds	45 812 (bp)	Number of protein-coding genes	12 260
Median size of scaffolds	33 955 (bp)	Mean protein size	483 (aa)
Main functional annotation			
General-content databases		Domain-specific databases	
Pfam	6965	dbCAN	369
InterPro	8186	MEROPS	382
eggNOG	9237		
Additional functional features			
Proteins with signal peptides	1093	Proteins with transmembrane helices	2585

## 2 Experimental design, materials, and methods

### 2.1 Fungal strain isolation and genetic verification

The fungal strain of *Steccherinum ochraceum* (Pers. ex J.F. Gmel.) Gray was isolated (August 01, 2013) from basidiospores collected from a fallen dry aspen branch in the polydominant temperate deciduous broadleaf forest (Kaluzhskiye Zaseki Nature Reserve, Russia; N 53º33′28.4″; E 35º38′24.4″). After morphological and genetic verifications, the strain was deposited in the Komarov Botanical Institute Basidiomycetes Culture Collection (LE-BIN; St. Petersburg, Russia) as *S. ochraceum* LE-BIN 3174.

For the genetic verification, the genomic DNA (gDNA) was extracted as described later in the “Genomic DNA Isolation, Library Preparation and Sequencing” section of this manuscript, and the sequence of ITS1-5.8S rRNA-ITS2 region was obtained using the standart primers: ITS1F 5′–CTT GGT CAT TTA GAG GAA GTA A–3′ and ITS4B 5′–CAG GAG ACT TGT ACA CGG TCC AG–3′. The PCR amplification was performed using the Encyclo PCR kit (Evrogen, Russia) under the following conditions: 1 cycle of 5 min at 95 °C; 25 cycles of 1 min at 90 °C, 1 min at 56 °C, and 1 min at 72 °C; 1 cycle of 10 min at 72 °C. Obtained PCR reaction mixture was resolved using 1,2% agarose gel. The performed PCR amplification produced the single PCR-product with approximate length of 830 bp. The obtained product was ceased from the gel and purified with QIAquick Gel Extraction Kit (Qiagen, USA), according to the manufacturer's instructions. The Sanger sequencing of the obtained fragment was performed at the Evrogen JSC (Russia, Moscow).

### 2.2 Genomic DNA isolation, Library Preparation and Sequencing

For gDNA extraction, *S. ochraceum* LE-BIN 3174 was statically cultivated at 26–28 °C in 750-mL Erlenmeyer flasks contained 200 mL of glucose-peptone (GP) medium (per 1 L of dH_2_O): 3.0 g peptone, 10.0 g glucose, 0.6 g KH_2_PO_4_, 0.4 g K_2_HPO_4_, 0.5 g MgSO_4_, 50 mg MnSO_4_, 1 mg ZnSO_4_ and 0.5 mg FeSO_4_. The mycelium was ground in liquid nitrogen, and gDNA was extracted using DNeasy Plant Mini Kit (Qiagen, US). The quality and quantity of the isolated DNA were checked using Agilent Bioanalyzer 2100 (Agilent Technologies, US) and Qubit fluorimeter (Thermo Fisher Scientific, US).

After ultrasonic fragmentation the gDNA was prepared for sequencing using TruSeq DNA Sample Prep Kit (Illumina, US). The quality and quantity of the obtained DNA-library were checked using Agilent Bioanalyzer 2100 and StepOnePlus Real-Time PCR System (Thermo Fisher Scientific, US). The whole genome sequencing was carried out with Illumina HiSeq 2500 system (Illumina, US) using HiSeq Rapid SBS Kit v2 at the Evrogen JSC (Russia, Moscow).

### 2.3 Genome sequencing, assembly and annotation

The shotgun sequencing produced 2 × 47,868,586 paired-end reads (2 × 100 bp) with an insert size of 300–500 bp. The reads were further processed with CLC Genomics Workbench 11.0 (Qiagen, US) as follows: (1) adapters were removed from all reads; (2) all reads were trimmed based on their quality; (3) reads were sampled to reduce coverage to a maximum average coverage of 100 × ; (4) reads were *de novo* assembled, and resulted contigs were scaffolded.

Genome structural and functional annotations were performed using Funannotate pipeline v1.5.0 (https://github.com/nextgenusfs/funannotate).

The structural annotation step included: (1) repeat masking with the RepeatMasker package (http://www.repeatmasker.org/) using the RepBase repeats libraries [3]; (2) *ab initio* protein-coding gene prediction with self-trained GeneMark-ES [4] and AUGUSTUS [5] trained using BUSCO 2.0 [6] gene models (*Phanerochaete chrysosporium* was selected as a closely-related species); (3) *ab initio* tRNA-coding gene prediction with tRNAscan-SE [7]; (4) integration and filtering of the obtained gene models.

The functional annotation of the predicted protein-coding genes was performed with three general-content databases: the protein families database – Pfam [8], the integrative protein signature database – InterPro [9], and the orthologous groups database – eggNOG [10]. Additionally, two domain-specific databases were employed: carbohydrate-active enzyme (CAZyme) database – dbCAN [11], and peptidase database – MEROPS [12]. The prediction of transmembrane topologies and signal peptides was performed with Phobius [13] and SignalP [14], respectively.

The data on genome sequencing, assembly and annotation are presented in Table 1.

As a result of general functional prediction, 6019 genese were annotated with the GO terms. In total, 10,648 GO terms were assigned, from which 1707 were GO terms related to “Cellular component” class, 5207 – to “Molecular function” class, and 3734 – to “Biological process” class (Fig. 1).

### 2.4 The peculiarities of the S. ochraceum LE-BIN 3174 CAZymes genome content

Based on the sequenced genome, the CAZymes repertoire of *S. ochraceum*LE-BIN 3174 was inferred and compared with those of the 8 fungi belonging to the different ecological niches and trophic groups. From the Polyporales order: *Trametes versicolor*, *Trametes pubescens*, and *Trametes hirsuta* (all are primary colonizers on *lignum*). From the Agaricales order: *Gymnopilus junonius* (secondary colonizer on *lignum*), *Hymenopellis radicata* (deep root mushroom, *lignum*), *Mycena galopus* (saprotroph on *folia dejecta*), *Crucibulum laeve* (saprotroph on *stramentum*), and *Agrocybe praecox* (saprotroph on *humus*).

Comparison of the total CAZymes contentis present in Fig. 2.

Comparison of the content of CAZymes acting on different polymeric components of lignocellulose [15] is presented in Fig. 3. Please note, that the numbers do not add up properly due to the redundancy in the classification scheme that was advanced to reflect different enzymatic activities possessed by fungi rather than different CAZymes, since the same CAZyme can simultaneously act on several components of lignocellulose.
